# Efficacy and Safety of Benmelstobart Plus Anlotinib in Advanced Endometrial Cancer: Results of a Multi‐Cohort, Phase II Study (ETER200)

**DOI:** 10.1002/mco2.71081

**Published:** 2026-10-21

**Authors:** Shanhui Liang, Xiaojun Chen, Ke Wang, Dong Wang, Ruifang An, Jianqing Hou, Ying Yang, Danbo Wang, Pengpeng Qu, Bingzhong Zhang, Wei Duan, Xiumin Li, Aimin Zang, Guohua Yu, Hanmei Lou, Desheng Yao, Shuzhen Wang, Dong Yan, Liang Li, Xiaowei Liu, Weidong Zhao, Zhengzheng Chen, Jing Fei, Jundong Li, Jing Wang, Zhilian Wang, Shuzhong Yao, Fangling Ning, Huaijun Zhou, Li Sun, Lingya Pan, Qing Liu, Haihua Yang, Li Wang, Mingjun Zhang, Xiaoxiang Chen, Jianlin Yuan, Xinwen Zhang, Youzhong Zhang, Qinping Liao, Xiaohua Wu

**Affiliations:** ^1^ Department of Gynecologic Oncology Fudan University Shanghai Cancer Center Shanghai China; ^2^ Department of Gynecology and Oncology Obstetrics & Gynecology Hospital of Fudan University Shanghai China; ^3^ Department of Obstetrics and Gynecology Shanghai Tenth People's Hospital Shanghai China; ^4^ Department of Gynecologic Oncology Tianjin Cancer Hospital Tianjin China; ^5^ Gynecologic Oncology Center Chongqing University Cancer Hospital Chongqing China; ^6^ Department of Obstetrics and Gynecology First Affiliated Hospital of Xi'an Jiaotong University Xi'an China; ^7^ Department of Gynecology Yantai Yuhuangding Hospital Yantai China; ^8^ Fifth Department of Obstetrics and Gynecology Liaoning Cancer Hospital & Institute Shenyang China; ^9^ Department of Gynecology and Oncology Tianjin Central Hospital of Gynecology Obstetrics Tianjin China; ^10^ Department of Gynecology and Oncology Sun Yat‐Sen Memorial Hospital Sun Yat‐Sen University Guangzhou China; ^11^ Department of Gynecology and Oncology Beijing Obstetrics and Gynecology Hospital Capital Medical University Beijing China; ^12^ Department of Gynecology Linyi Cancer Hospital Linyi China; ^13^ Department of Medical Oncology Affiliated Hospital of Hebei University Baoding China; ^14^ Department of Oncology Weifang People's Hospital Weifang China; ^15^ Department of Gynecologic Oncology and Radiation Therapy Zhejiang Cancer Hospital Hangzhou China; ^16^ Department of Gynecology and Oncology Guangxi Medical University Cancer Hospital Nanning China; ^17^ Department of Obstetrics and Gynecology Beijing Chao‐Yang Hospital Capital Medical University Beijing China; ^18^ Department of Oncology Beijing Luhe Hospital Capital Medical University Beijing China; ^19^ Department of Oncology Meizhou People's Hospital Meizhou China; ^20^ Department of Oncology Affiliated Hospital of Jining Medical University Jining China; ^21^ Department of Obstetrics and Gynecology The First Affiliated Hospital of USTC Anhui Provincial Hospital Hefei China; ^22^ Department of Medical Oncology The First Affiliated Hospital of Shihezi University School of Medicine Shihezi China; ^23^ Department of Gynecology Sun Yat‐Sen University Cancer Center Guangzhou China; ^24^ Fifth Department of Gynecologic Oncology Hunan Cancer Hospital Changsha China; ^25^ Department of Obstetrics and Gynecology Second Hospital of Shanxi Medical University Taiyuan China; ^26^ Department of Obstetrics and Gynecology The First Affiliated Hospital Sun Yat‐Sen University Guangzhou China; ^27^ Department of Oncology Binzhou Medical University Hospital Binzhou China; ^28^ Department of Obstetrics and Gynecology Nanjing Drum Tower Hospital Nanjing China; ^29^ Department of Gynecologic Oncology Qingdao Central Medical Group Qingdao China; ^30^ Department of Obstetrics and Gynecology Chinese Academy of Medical Sciences and Peking Union Medical College Beijing China; ^31^ Department of Gynecology Gansu Provincial Maternity and Child‐Care Hospital Lanzhou China; ^32^ Department of Radiation Oncology Taizhou Hospital of Zhejiang Province Taizhou China; ^33^ Department of Gynecology and Oncology Henan Cancer Hospital Zhengzhou China; ^34^ Department of Oncology The Second Hospital of Anhui Medical University Hefei China; ^35^ Department of Gynecology and Oncology Jiangsu Cancer Hospital Nanjing China; ^36^ Department of Gynecology Affiliated Tumor Hospital of Xinjiang Medical University Urumqi China; ^37^ Department of Obstetrics and Gynecology Xi'an People Hospital (Xi'an Fourth Hospital) Xi'an China; ^38^ Department of Gynecology Qilu Hospital of Shandong University Jinan China; ^39^ Department of Obstetrics and Gynecology Beijing Tsinghua Changgung Hospital Beijing China

**Keywords:** advanced endometrial cancer, anti‐angiogenic therapy, immunotherapy, MSI/MMR status

## Abstract

Patients with advanced endometrial cancer (EC) have a poor prognosis and limited therapeutic options after failure of prior systemic therapy. In this multi‐cohort Phase II study (ETER200), patients with metastatic or recurrent EC who had received at least one chemotherapy regimen were enrolled. Based on MSI/MMR status, patients received benmelstobart plus anlotinib (Cohort 1), benmelstobart (Cohort 2), or anlotinib (Cohort 3) every 3 weeks. Primary endpoint was objective response rate (ORR) assessed by independent review committee (IRC) per RECIST v1.1. Here, the results of Cohort 1 expansion stage were reported. As of May 9, 2024, 85 of the 107 non‐MSI‐H/pMMR EC patients enrolled in Cohort 1 constituted the expansion stage population for primary efficacy analysis. The IRC‐assessed ORR was 34.1% (95% CI, 24.2–45.2), with a median duration of response of 15.2 months (95% CI, 6.9–not evaluable). Median progression‐free and overall survival were 8.8 months (95% CI, 5.8–15.2) and 21.8 months (95% CI, 19.5–29.1), respectively. Grade ≥ 3 treatment‐related adverse events occurred in 69.2% of patients, most commonly hypertension (21.2%) and palmar‐plantar erythrodysesthesia syndrome (9.4%). Overall, benmelstobart plus anlotinib showed promising anti‐tumor activity and an acceptable safety profile in advanced non‐MSI‐H/pMMR EC, supporting further evaluation of this combination in randomized Phase III studies.

Trial Registration: ClinicalTrials.gov, NCT04574284.

## Graphical abstract

The multi‐cohort, Phase II ETER200 study evaluated benmelstobart plus anlotinib or either agent alone in previously treated metastatic or recurrent endometrial cancer across molecular subtypes. In non–MSI‐H/pMMR tumors, benmelstobart plus anlotinib demonstrated durable antitumor activity without unexpected safety signals. These results supported its approval as the first PD‐L1 inhibitor plus tyrosine kinase inhibitor regimen for this population in China.

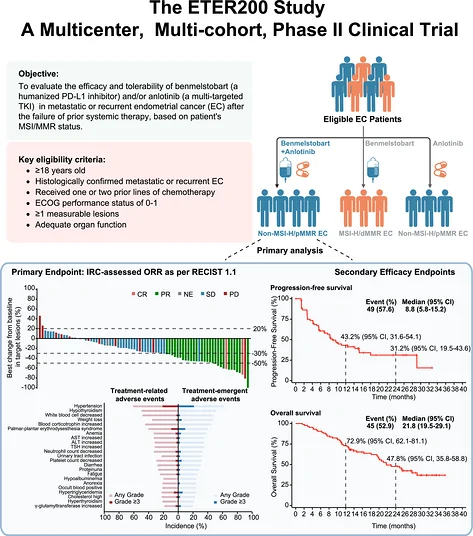

## 1 Introduction

Endometrial cancer (EC) is the second most common gynecologic malignancy worldwide, with both increasing incidence and mortality [1]. Approximately 30% of EC cases are characterized by microsatellite instability‐high/mismatch repair‐deficient (MSI‐H/dMMR), whereas the other 70% are non‐MSI‐H/mismatch repair‐proficient (pMMR) [2]. For patients with advanced or recurrent disease, treatment options are limited and long‐term outcomes remain poor regardless of histologic subtype, with a 5‐year survival rate of approximately 17% [2, 3]. In the era of biomarker‐directed therapy, a deeper understanding of MMR status in this disease has led to development of divergent second‐line strategies.

Immunotherapy with or without anti‐angiogenic therapy has been recommended as standard second‐line therapy for advanced EC according to molecular subtype [4]. However, the benefit of immunotherapy monotherapy is limited in patients with dMMR EC, with an objective response rate (ORR) ranging from 27% to 50% [5, 6, 7]. For more prevalent pMMR tumors, only two anti‐PD‐1‐based combinations (pembrolizumab plus lenvatinib, fruquintinib plus sintilimab) have been investigated and received region‐specific approval, demonstrating a modest ORR of about 32% and median progression‐free survival (PFS) of around 7 months [8, 9, 10]. Nevertheless, these existing regimens raised concerns about the risk–benefit balance, particularly for anti‐PD‐1‐based combinations due to frequent discontinuation (∼30%) and immune‐related toxicity (∼70%) [8, 9, 10]. In this context, PD‐L1 inhibitors may be an important possibility to address this unmet need since they directly bind to PD‐L1 on tumor cells, potentially enhancing efficacy while reducing toxicity [11]. Clinical evidence also demonstrates fewer Grade ≥ 3 treatment‐related adverse events (TRAEs) with PD‐L1 inhibitors than with PD‐1 inhibitors [12]. Benmelstobart is a novel humanized IgG1 PD‐L1 inhibitor with a genetically modified Fc domain to minimize FcγR binding [13]. This feature may offer favorable safety profile for benmelstobart over other PD‐(L)1 inhibitors, as evidenced by a lower incidence of serious events (27.5% vs. 39%–44%) [13, 14, 15]. More importantly, direct comparative studies provide robust evidence that benmelstobart‐containing combinations achieve efficacy and tolerability comparable to or exceeding that observed with PD‐1 inhibitors [16, 17]. Given this premise, benmelstobart may be an attractive option in advanced EC, regardless of MMR status.

Anlotinib is an oral, multi‐targeted tyrosine kinase inhibitor (TKI) with a broader target spectrum and superior anti‐angiogenic activity versus other TKIs (i.e., sunitinib, sorafenib, and nintedanib) [18, 19, 20], which makes it an ideal partner for PD‐(L)1 inhibitors. It has shown clinical activity with manageable toxicity in combination regimens for EC [21, 22]. Extensive preclinical evidence supporting the synergy of anlotinib plus PD‐(L)1 inhibitors also provides a rationale for combining anlotinib with benmelstobart, in which anlotinib facilitates vessel normalization, contributes to drug delivery and distribution, and overcomes the immunosuppressive microenvironment [23, 24, 25]. Multiple large‐scale Phase III trials have established the efficacy and safety benefits of this combination in various solid tumors [16, 17, 26, 27], further supporting its possibility as a better‐tolerated option.

Building upon prior results, we conducted a multi‐cohort, Phase II study (ETER200) to investigate the efficacy and safety of benmelstobart plus anlotinib, or each agent alone, in metastatic or recurrent EC after the failure of prior systemic therapy, with patients enrolled into specific cohorts according to MSI/MMR status. Based on the encouraging results of this study, benmelstobart plus anlotinib has been approved for the second‐line treatment of advanced non‐MSI‐H/dMMR EC. Herein, we present the results of Cohort 1 evaluating this combination in patients with advanced non‐MSI‐H/pMMR EC. In this study, benmelstobart plus anlotinib showed promising and durable antitumor activity with an acceptable safety profile. These findings support further evaluation of this combination and provide additional evidence for PD‐L1 inhibition plus anti‐angiogenic therapy as a treatment strategy in previously treated non‐MSI‐H/pMMR EC.

## 2 Results

### 2.1 Patient Disposition and Characteristics

Between September 29, 2020, and May 9, 2023, of 264 patients assessed for eligibility, 170 were enrolled from 38 participating centers in China (Table S1), including 107 in Cohort 1, 32 in Cohort 2, and 31 in Cohort 3 (Figure 1). The present analysis of efficacy and safety focused on the 107 patients with non‐MSI‐H/pMMR EC from Cohort 1.

**FIGURE 1:**
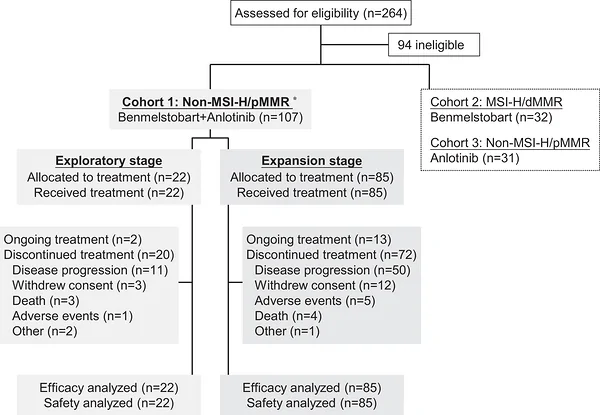
Overall study design and Cohort 1 patient disposition. *Cohort 1 data are reported in the present analysis. dMMR, mismatch repair‐deficient; MSI‐H, microsatellite instability‐high; pMMR, mismatch repair‐proficient.

Cohort 1 initially enrolled 22 patients into the exploratory stage (Table S2), of whom 8 (36.4%) patients achieved an objective response (Table S3) and 14 (63.6%) experienced Grade ≥ 3 TRAEs, with no treatment‐related deaths (Table S4). Additional data from the exploratory stage are listed in Figure S1 and Tables S4–S6. After meeting the predefined criteria for continued accrual and demonstrating an acceptable safety profile, Cohort 1 proceeded to the expansion stage, with an additional 85 patients enrolled, which comprised the primary efficacy analysis population. Together, these patients and the initial exploratory cohort comprised the safety analysis population.

At the data cutoff (May 9, 2024), 20 (90.9%) of 22 patients in the exploratory stage and 72 (84.7%) of 85 patients in the expansion stage had discontinued the study treatment; treatment is ongoing in 2 (9.1%) and 13 (15.3%) patients, respectively. The most common reason for treatment discontinuation in both stages was disease progression. At the time of analysis, the median follow‐up was 37.5 months (confidence interval [CI], 35.3–39.1) for patients in the exploratory stage and 27.8 months (95% CI, 25.5–30.2) for those in the expansion stage.

Baseline characteristics of the 85 patients in the expansion stage are summarized in Table 1. All patients had received at least one prior line of systemic therapy. Of these 85 patients, most had undergone prior radiotherapy (50 [58.8%]) or pelvic surgery (82 [96.5%]). Patient characteristics in the expansion stage (*n* = 85) were representative of those in the overall Cohort 1 population (*n* = 107; Table S2).

**TABLE 1:** Baseline characteristics of patients.

Characteristics	Patients (*n* = 85)^a^
Age, years	60 (30–78)
< 65 years	63 (74.1)
≥ 65 years	22 (25.9)
Histology type at diagnosis	
Endometrioid carcinoma	68 (80.0)
Serous carcinoma	16 (18.8)
Other	1 (1.2)
ECOG performance status	
0	30 (35.3)
1	54 (63.5)
Missing	1 (1.2)
FIGO stage at diagnosis^b^	
I	23 (27.1)
II	5 (5.9)
III	26 (30.6)
IV	28 (32.9)
Missing	3 (3.5)
Disease status	
Recurrent	74 (87.1)
Metastatic	85 (100.0)
Metastatic sites	
Liver	27 (31.8)
Brain	4 (4.7)
Prior lines of systemic treatment	
1	69 (81.2)
2	16 (18.8)
Prior radiation therapy	50 (58.8)
Prior pelvic surgery	82 (96.5)
Treatment‐free interval^c^	
≤ 6 months	51 (60.0)
> 6 months	34 (40.0)

*Note*: Data are median (range) or *n* (%).

Abbreviations: ECOG, Eastern Cooperative Oncology Group; FIGO, International Federation of Gynecology and Obstetrics.

^a^ Data of the patients in Cohort 1 expansion stage are shown.

^b^ FIGO stage was determined as per FIGO 2009 staging system, except for five patients staged per FIGO 1971 staging system.

^c^ Treatment‐free interval was defined as the interval between the end of the last prior antitumor therapy and the initiation of second‐line therapy.

### 2.2 Efficacy Outcomes

Among 85 non‐MSI‐H/pMMR patients who received benmelstobart plus anlotinib, the independent review committee (IRC)‐assessed ORR was 34.1% (95% CI, 24.2–45.2; Table 2), including 2 (2.4%) patients with complete response (CR) and 27 (31.8%) with partial response (PR). An additional 37 (43.5%) patients had stable disease (SD), resulting in a disease control rate (DCR) of 77.6% (95% CI, 67.3–86.0). Most patients (63/85 [74.1%]) had a decrease in target lesions from baseline, including 14 (16.5%) with a reduction ≥ 50% (Figure 2A). Of 29 responders, 19 (65.5%) achieved a rapid response within 3 months and 23 (79.3%) have maintained responses for at least 6 months, with the longest response ongoing at 2 years (Figure 2B). Longitudinal change in tumor size from baseline is shown in Figure 2C. As per IRC assessment, median duration of response (DoR) was 15.2 months (95% CI, 6.9–not evaluable), with a 6‐month DoR rate of 78.3% (95% CI, 57.9–89.6; Figure S2). Objective responses across subgroups were generally consistent among patients with different baseline characteristics (Figure S3). At data cutoff, 36 patients did not have disease progression, and 40 remained alive. The median IRC‐assessed PFS was 8.8 months (95% CI, 5.8–15.2; Figure 3A), with estimated 12‐month and 24‐month PFS rates of 43.2% (95% CI, 31.6–54.1) and 31.2% (95% CI, 19.5–43.6), respectively. Median overall survival (OS) was 21.8 months (95% CI, 19.5–29.1; Figure 3B). The estimated 12‐month and 24‐month OS rates were 72.9% (95% CI, 62.1–81.1) and 47.8% (95% CI, 35.8–58.8), respectively. The results in the overall Cohort 1 population (*n* = 107) were consistent with those observed in the primary analysis of 85 patients (Figure S4 and Table S3). Investigator‐assessed efficacy endpoints are shown in Figure S5 and Table S3, and were similar to those observed by IRC assessment.

**TABLE 2:** Independent review committee‐assessed response to treatment.

Response^b^	Patients (*n* = 85)^a^
Objective response	
No. of patients	29
% (95% CI)	34.1 (24.2–45.2)
Best response, no. (%)	
Complete response	2 (2.4)
Partial response	27 (31.8)
Stable disease	37 (43.5)
Progressive disease	13 (15.3)
Not evaluable^c^	6 (7.1)
Median DoR (95% CI), months	15.2 (6.9–NE)
6‐month DoR, % (95% CI)	78.3 (57.9–89.6)
Disease control rate, % (95% CI)	77.6 (67.3–86.0)

Abbreviations: CI, confidence interval; DoR, duration of response; NE, not evaluable.

^a^ Data of the patients in Cohort 1 expansion stage are shown.

^b^ Analyses were based on confirmed response as per RECIST version 1.1.

^c^ Patients without confirmed response or post‐baseline tumor assessment.

**FIGURE 2:**
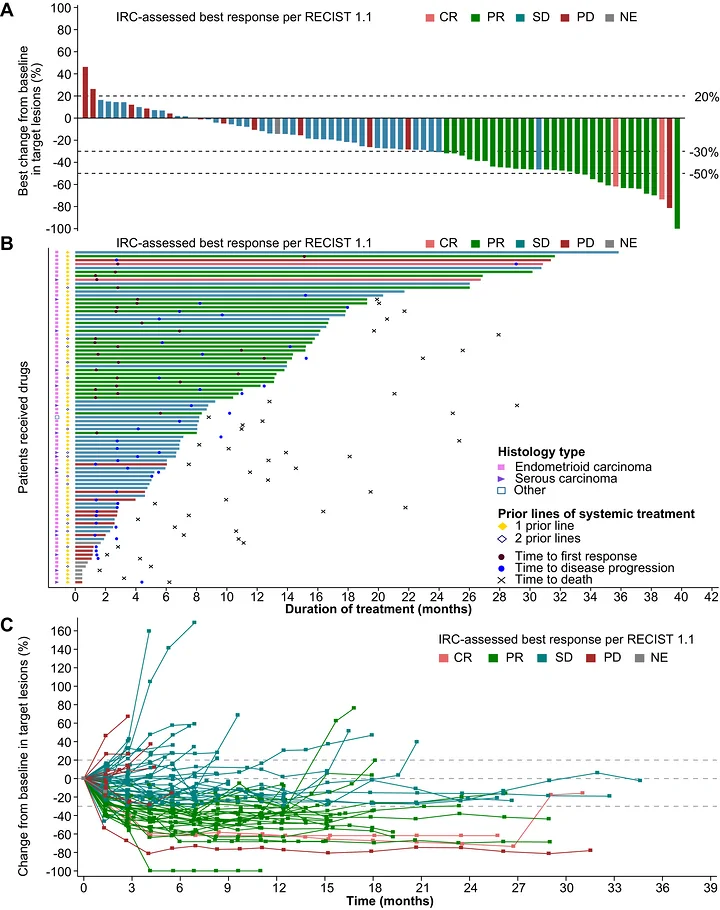
Tumor response assessed by IRC per RECIST 1.1 in the expansion stage (*n* = 85). (A) Waterfall plot of best percentage change in tumor size from baseline. (B) Swimmer plot of time on study and duration of responses. (C) Longitudinal percentage change in tumor size from baseline over time. NE indicates patients without confirmed response or post‐baseline tumor assessment. CR, complete response; IRC, independent review committee; MSI‐H, microsatellite instability‐high; PD, disease progression; NE, not evaluable; pMMR, mismatch repair‐proficient; PR, partial response; RECIST 1.1, Response Evaluation Criteria in Solid Tumors version 1.1; SD, stable disease.

**FIGURE 3:**
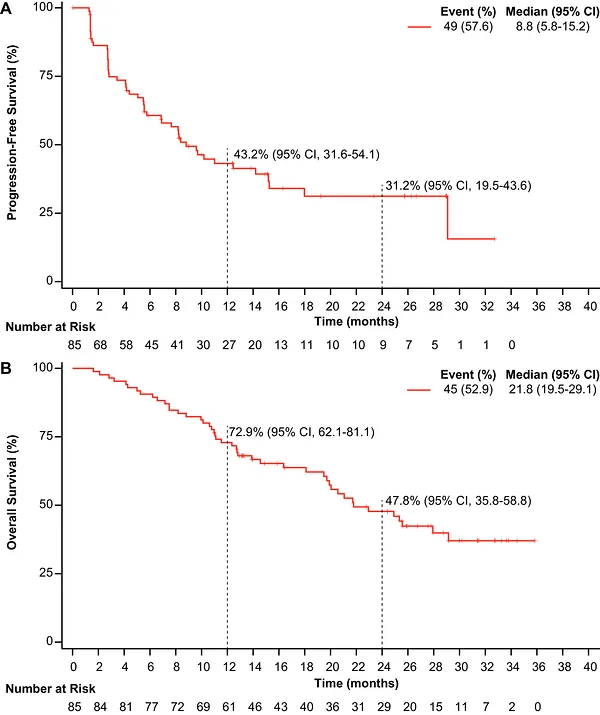
Kaplan–Meier plots for survival in the expansion stage (*n* = 85). (A) Kaplan–Meier plot for independent review committee‐assessed progression‐free survival; (B) Kaplan–Meier plot for overall survival.

In the post hoc exploratory analysis, patients in the primary efficacy population were stratified by treatment‐free interval (TFI; ≤ 6 months vs. > 6 months). Based on IRC assessment, the ORR was 38.2% in the TFI > 6 months subgroup and 31.4% in the TFI ≤ 6 months subgroup. Median PFS was 14.2 months and 6.9 months, respectively (Table S7). Similar results were observed based on investigator assessment. As the analysis was not powered for statistical comparisons, these findings should be interpreted with caution.

### 2.3 Exposure and Safety

Data on treatment exposure and duration are detailed in Table S8. All patients in Cohort 1 (*n* = 107) received a median of 11 cycles (range, 1–49) of benmelstobart and a median duration of anlotinib treatment of 152.5 days (range, 14–770). Among 85 patients in the expansion stage, 84 (98.8%) reported at least one treatment‐emergent adverse event (TEAE) of any grade, with 77.6% of Grade ≥ 3 severity (Table 3). Common Grade ≥ 3 TEAEs included hypertension (21.2%) and palmar‐plantar erythrodysesthesia syndrome (9.4%). Thirty‐five (41.2%) of 85 patients experienced serious TEAEs (Table S9). TEAEs led to dose reductions of any drug in 56 (65.9%) patients; dose interruptions in 34 (40.0%) patients; and permanent discontinuation in 10 (11.8%) patients.

**TABLE 3:** Adverse events in the expansion stage (*n* = 85).

	Treatment‐emergent adverse events		Treatment‐related adverse events	
Adverse events (%)^a^	Any grade	Grade ≥ 3	Any grade	Grade ≥ 3^b^
Any TRAEs	84 (98.8)	66 (77.6)	83 (97.6)	60 (70.6)
Frequent TRAEs (≥ 20% incidence)				
Hypertension	52 (61.2)	18 (21.2)	52 (61.2)	18 (21.2)
Hypothyroidism	43 (50.6)	0	42 (49.4)	0
White blood cell decreased	41 (48.2)	2 (2.4)	41 (48.2)	2 (2.4)
Blood corticotrophin increased	38 (44.7)	0	38 (44.7)	0
Palmar‐plantar erythrodysesthesia syndrome	34 (40.0)	8 (9.4)	34 (40.0)	8 (9.4)
Weight loss	40 (47.1)	4 (4.7)	30 (35.3)	2 (2.4)
TSH increased	29 (34.1)	0	27 (31.8)	0
AST increased	30 (35.3)	2 (2.4)	28 (32.9)	2 (2.4)
Diarrhea	26 (30.6)	2 (2.4)	24 (28.2)	2 (2.4)
ALT increased	29 (34.1)	2 (2.4)	27 (31.8)	2 (2.4)
Neutrophil count decreased	28 (32.9)	3 (3.5)	28 (32.9)	3 (3.5)
Anemia	32 (37.6)	3 (3.5)	28 (32.9)	3 (3.5)
Platelet count decreased	27 (31.8)	4 (4.7)	25 (29.4)	3 (3.5)
Proteinuria	24 (28.2)	2 (2.4)	23 (27.1)	2 (2.4)
Hypertriglyceridemia	20 (23.5)	5 (5.9)	19 (22.4)	5 (5.9)
Cholesterol high	19 (22.4)	2 (2.4)	19 (22.4)	2 (2.4)
Fatigue	22 (25.9)	2 (2.4)	21 (24.7)	2 (2.4)
Hyperthyroidism	18 (21.2)	0	18 (21.2)	0
Urinary tract infection	27 (31.8)	2 (2.4)	19 (22.4)	2 (2.4)
Anorexia	20 (23.5)	2 (2.4)	18 (21.2)	0
Hypoalbuminemia	21 (24.7)	0	17 (20.0)	0
Occult blood positive	20 (23.5)	0	18 (21.2)	0
γ‐glutamyltransferase increased	17 (20.0)	3 (3.5)	16 (18.8)	3 (3.5)

Abbreviations: ALT, alanine aminotransferase; AST, aspartate aminotransferase; TRAEs, treatment‐related adverse events; TSH, thyroid‐stimulating hormone.

^a^ Data are the number (%) of patients with an adverse event.

^b^ In Cohort 1, one patient died from large intestine perforation, and one died from lower gastrointestinal hemorrhage; both events were considered to be possibly related to anlotinib.

Overall, 97.6% of 85 patients experienced adverse events assessed by the investigator as possibly related to treatment (Table 3), commonly including hypertension (61.2%), hypothyroidism (49.4%), decreased white blood cell (48.2%), and increased blood corticotrophin (44.7%). Grade ≥ 3 TRAEs were reported in 60 (70.6%) patients, the most frequent being hypertension (21.2%) and palmar‐plantar erythrodysesthesia syndrome (9.4%). Serious TRAEs were reported in 23.5% of patients. TRAEs led to dose interruption, reduction, and discontinuation in 48 (56.5%), 34 (40.0%), and 9 (10.6%) patients, respectively. Immune‐related AEs (irAEs) were reported in 58.8% of patients, leading to benmelstobart interruption in 16.8% of patients (Table S9). Hypothyroidism (31.8%) was the most common irAE (Table S10).

## 3 Discussion

To the best of our knowledge, ETER200 represents the first trial investigating the combination of a PD‐L1 inhibitor and TKI in patients with previously treated advanced non‐MSI‐H/pMMR EC. In this challenging disease setting, benmelstobart plus anlotinib demonstrated deep and durable responses, with an ORR of 34.1% and a median DoR of 15.2 months, which translated into encouraging survival outcomes (median PFS 8.8 months; median OS 21.8 months). This combination also demonstrated an acceptable safety profile, with no new safety signals. Supported by findings from ETER200, benmelstobart plus anlotinib has become the first PD‐L1 inhibitor plus TKI regimen approved in China for previously treated advanced non‐MSI‐H/pMMR EC [28].

Over the past decade, several efforts have been devoted to improving outcomes for patients with previously treated, advanced non‐MSI‐H/pMMR EC by combining anti‐angiogenic agents with immune checkpoint inhibitors (ICIs) [8, 9, 10, 29]. Based on positive results of the KEYNOTE‐775 trial, pembrolizumab plus lenvatinib has been recommended as the new standard second‐line treatment in this disease, demonstrating an ORR of 32.4%, a median DoR of 12.9 months, a median PFS of 6.7 months, and a median OS of 18.0 months [9]. In the ETER200 trial, benmelstobart plus anlotinib demonstrated encouraging and durable antitumor activity, with an ORR of 34.1% and a median DoR of 15.2 months, along with favorable survival outcomes, regarding median PFS and OS of 8.8 and 21.8 months, respectively. In a recently published report, an anti‐PD‐1‐based combination of sintilimab and fruquintinib also showed comparable outcomes in a similar population, with respect to ORR (31.6%), PFS (7.1 months), and OS (21.8 months) [10]. Given inherent limitations of cross‐trial comparisons, differences in study design, patient selection, prior therapies, and geographic regions should be considered when interpreting our results. The relatively young age of the study population (< 65 years, 74.1%), the high proportion of patients receiving only one prior line of therapy (81.2%), prior pelvic surgery (96.5%), and prior radiotherapy (58.8%) may have contributed to the promising outcomes observed in our study. In particular, the predominance of less‐aggressive endometrioid carcinoma (80%), which is generally associated with a favorable prognosis [30], might be another consideration and may limit the generalizability of the findings to broader patient populations. Furthermore, the broad‐spectrum anti‐angiogenic activity of anlotinib [19, 20], the high affinity and stability of Fc‐engineered IgG1 anti‐PD‐L1 benmelstobart [31], and their synergistic interaction may also partly account for the observed favorable outcomes [23, 24, 25]. Supporting this hypothesis, prior clinical evidence suggests that anlotinib may sensitize tumors to ICIs [21], and preclinical data indicate that anlotinib enhances retention and distribution of ICIs within the tumor [25]. Collectively, these findings support the antitumor activity of benmelstobart plus anlotinib in this setting, warranting further validation in broader patient populations.

The safety profile of benmelstobart with or without anlotinib in the ETER200 trial was consistent with that of individual agents in other tumor types [13, 32, 33], with no new safety signals identified. Although common or serious adverse events occurred more frequently with benmelstobart plus anlotinib than previously reported for individual agents [13, 32], this was an anticipated consequence of the combination strategy and longer treatment duration. The overall incidence of Grade ≥ 3 TRAEs (70.6%) and serious TRAEs (23.5%) still warrants careful attention, underscoring the need for close monitoring and supportive care during treatment. Notably, the most frequent Grade ≥ 3 TRAE was hypertension (21.2%), a well‐recognized class effect of anti‐angiogenic TKIs. Interestingly, post hoc analyses have suggested that treatment‐emergent hypertension may be associated with improved antitumor efficacy of anti‐angiogenic agents [34, 35], although this association remains exploratory and should be interpreted with caution. Most toxicities were resolved with dose modifications, including dose interruptions (56.5%) and dose reductions (40.0%), resulting in permanent treatment discontinuation due to TRAEs in only 10.6% of patients. Although dose modifications may increase treatment burden, most patients remained on treatment and derived benefit from study treatment. Previous studies have also reported treatment discontinuation due to TRAEs in 31.5% of patients receiving pembrolizumab plus lenvatinib and 15.8% of those receiving fruquintinib plus sintilimab [9, 10]. As expected, our anti‐PD‐L1‐based combination was associated with a lower incidence of irAEs compared to prior anti‐PD‐1‐based combinations (58.8% vs. 67.2%–75.5%), particularly for the most frequent thyroid irAEs (e.g., hypothyroidism [31.8% vs. 45.9%–57.6%] and hyperthyroidism [3.5% vs. 11.6%–25.5%]). This is particularly important, as thyroid irAEs are often permanent and require replacement therapy, which may adversely affect treatment adherence and long‐term quality of life [36]. Overall, benmelstobart plus anlotinib was generally safe in advanced EC.

Several limitations of the ETER200 study should be acknowledged. First, ETER200 was a single‐arm, multi‐cohort, non‐randomized trial conducted exclusively in Chinese patients, without an active comparator; therefore, efficacy results should be interpreted with caution. Of note, the ongoing confirmatory Phase III trial (NCT06475599) will further validate the efficacy of this combination. Second, consistent with contemporary pivotal immunotherapy trials in EC, our study used MMR/MSI status for patient selection, while additional biomarker analyses (e.g., PD‐L1 expression, *POLE* and *TP53* mutation status, or molecular classification according to TCGA/ProMisE) were not pre‐planned. This limited further evaluation of treatment efficacy across molecular subgroups and identification of patients more likely to benefit from the treatment, underscoring the need for future studies with comprehensive molecular profiling. Third, as ETER200 was initiated before the establishment of ICIs as standard first‐line therapy for advanced EC, no enrolled patients had received prior immunotherapy, limiting the findings’ generalizability to contemporary clinical practice. The absence of quality‐of‐life data is another limitation for the comprehensive assessment of treatment tolerability from the patient's perspective. Finally, our findings should be interpreted cautiously since the data on DoR and OS have yet to fully mature due to relatively short follow‐up periods; follow‐up is still ongoing.

In conclusion, benmelstobart combined with anlotinib demonstrated durable and clinically meaningful benefits, without unexpected safety signals, in patients with previously treated advanced non‐MSI‐H/pMMR EC, suggesting that it may represent a promising second‐line treatment option. These findings support further validation of benmelstobart plus anlotinib in randomized trials.

## 4 Materials and Methods

### 4.1 Study Design and Patients

ETER200 (ClinicalTrials.gov, NCT04574284) is a multi‐center, non‐randomized, multi‐cohort, open‐label, Phase II study conducted at 38 participating centers in China from September 29, 2020, to May 9, 2023. Patients were enrolled into three cohorts based on the MSI/MMR status: Cohort 1—non–MSI‐H/pMMR EC received benmelstobart plus anlotinib; Cohort 2—MSI‐H/dMMR EC received benmelstobart monotherapy; and Cohort 3—non–MSI‐H/pMMR EC received anlotinib. Cohort 1 consisted of two stages: an exploratory stage to assess safety and preliminary efficacy, and an expansion stage that depends upon exploratory results.

Adult women (≥ 18 years) with histologically confirmed metastatic or recurrent EC (relapsed or refractory to prior chemotherapy), who had received one or two prior lines of chemotherapy, had an Eastern Cooperative Oncology Group (ECOG) performance status of 0–1, at least one measurable lesion per Response Evaluation Criteria in Solid Tumors version 1.1 (RECIST v 1.1), a life expectancy of ≥ 3 months, and adequate organ function were eligible for enrollment. Available biopsy specimens for central MSI/MMR review were required for patients in Cohorts 1 and 2. Key exclusion criteria included prior exposure to anti‐angiogenic agents or ICIs for patients in Cohort 1, other active malignancies within 3 years before study entry, and uncontrolled diseases.

The study protocol and all amendments were approved by the institutional review board or independent ethics committees at each participating center (approval numbers are listed in Table S1) and relevant regulatory authorities. The trial was performed in accordance with the Declaration of Helsinki, Good Clinical Practice guidelines, and applicable local regulatory requirements. All patients provided written informed consent.

### 4.2 Procedure and Assessment

Each cohort enrolled patients independently and in parallel. Patients were assigned to each cohort based on the baseline MSI/MMR status (assessed by immunohistochemistry or polymerase chain reaction) at study entry. Patients in Cohort 1 (non–MSI‐H/pMMR EC) received benmelstobart plus anlotinib; patients in Cohort 2 (MSI‐H/dMMR EC) received benmelstobart monotherapy; and patients in Cohort 3 (non–MSI‐H/pMMR EC) received anlotinib monotherapy. Anlotinib was given at 12 mg orally on Days 1–14, and benmelstobart was given at 1200 mg intravenously on Day 1 in a 21‐day cycle, until confirmed disease progression, unacceptable toxicity, consent withdrawal, or investigator decision. Two steps of dose reduction for anlotinib were permitted (to 10 or 8 mg per day) if a dose reduction criterion was fulfilled. Anlotinib was allowed to be interrupted for up to 4 weeks. Treatment was discontinued in patients who had intolerable toxicity despite anlotinib dose adjustment. Dose reductions of benmelstobart were not permitted, but interruptions were allowed for up to 12 weeks to manage toxicity.

Radiological response was assessed by the IRC and investigators according to RECIST version 1.1. Radiological evaluation was performed by computed tomography or magnetic resonance imaging at screening, every 2 cycles for the first 18 cycles, and every 4 cycles thereafter until confirmed disease progression. Confirmation of PR or CR was required at the next assessment or at least 4 weeks later. If patients who received benmelstobart had RECIST v1.1‐defined disease progression, a second confirmation by immunotherapy RECIST (iRECIST) criteria was required. AEs were monitored from the first dose to 90 days after the last dose of study treatment. All AEs were assessed by investigators and classified by severity grade using the National Cancer Institute Common Terminology Criteria for Adverse Events (NCI‐CTCAE) version 5.0. The incidence and severity of TEAEs, TRAEs, serious AEs, and irAEs were assessed. The causality was determined by investigators as planned. Serious events were defined as those resulting in death, life‐threatening events, inpatient hospitalization, prolongation of existing hospitalization, a persistent/severe disability or incapacity, congenital anomalies/birth defects, or any other medically important events that required intervention. Survival assessments were performed every 2 months after the safety follow‐up period.

### 4.3 Endpoints

The primary endpoint was the IRC‐assessed ORR as per RECIST version 1.1. Key secondary endpoints included investigator‐assessed ORR, DCR, DoR, and PFS assessed by both IRC and investigator. Other secondary endpoints included the DoR rate, OS, and safety. ORR was defined as the percentage of patients with CR or PR. DCR was defined as the percentage of patients with CR, PR, or SD. DoR was defined as the time from the first documented response to PD or death from any cause among responders. PFS was defined as the time from treatment initiation to disease progression or death from any cause. OS was defined as the time from treatment initiation to death from any cause.

### 4.4 Statistical Analysis

Sample size estimation of Cohort 1 was based on a two‐stage design that allowed for early stopping for futility. In the exploratory stage, 22 patients would be enrolled. Based on historical controls, an ORR of 15% or less was not of interest for further clinical development. If there were eight or more objective responses (corresponding to a 95% CI lower limit of ORR > 15%), accrual would continue to the expansion stage. In the expansion stage, a total of 74 patients were planned for enrollment based on a target ORR of 30% to provide 90% power at a two‐sided significance level of 5%. Considering a dropout rate of 10%, 83 patients were required.

Efficacy was assessed in patients who received at least one dose of study treatment. Per the prespecified statistical analysis plan, the primary efficacy analysis population comprised patients enrolled in the expansion stage. Patients enrolled during the exploratory stage were analyzed separately as an exploratory cohort and were not included in the primary efficacy analysis. Safety was assessed in all treated patients from both the exploratory and expansion stages.

Descriptive statistics were used to summarize the baseline characteristics and safety data. Tumor response was summarized with 95% CIs by the Clopper‐Pearson method. For response analyses, patients without baseline or post‐baseline response assessments were considered non‐responders. Analyses of time‐to‐event endpoints (PFS, DoR, and OS) were performed using Kaplan–Meier methods. For PFS and DoR, patients who were alive without progression or lost to follow‐up were censored at the last assessment. For OS, patients who were alive or lost to follow‐up were censored at the last day known alive. A post hoc exploratory, descriptive analysis was conducted to explore the potential effects of TFI (defined as the interval between the end of the last prior antitumor therapy and the initiation of second‐line therapy) on ORR and PFS. Patients were categorized using a 6‐month TFI cutoff, based on previous evidence supporting its utility for stratifying outcomes in recurrent EC [37, 38]. All analyses were conducted using SAS software version 9.4 (SAS Institute, Cary, NC, USA).

## Author Contributions

X.H.W. conceived and designed the study, had full access to all of the data in the study, and took responsibility for the integrity of the data and the accuracy of the data analysis. X.H.W., S.H.L., X.J.C., K.W., D.W. and R.F.A. implemented and performed the experiments. X.H.W., S.H.L., X.J.C., K.W., J.Q.H., Y.Y., D.B.W., P.P.Q., B.Z.Z., W.D., X.M.L., A.M.Z., G.H.Y., H.M.L. and D.S.Y. analyzed the data and experimental results. X.H.W., S.H.L., S.Z.W., D.Y., L.L., X.W.L. and W.D.Z. drafted the paper. Z.Z.C., J.F., J.D.L., J.W., Z.L.W., S.Z.Y., F.L.N., H.J.Z., L.S., L.Y.P., Q.L., H.H.Y., L.W. M.J.Z., X.X.C., J.L.Y., X.W.Z., Y.Z.Z. and Q.P.L. reviewed the paper. All authors read and approved the final version of the manuscript.

## Funding

This work was sponsored by the Chia Tai Tianqing Pharmaceutical Group Co. Ltd.

## Ethics Statement

The study protocol and all amendments were approved by the institutional review board or independent ethics committees at each participating center before patient enrollment (Lead site: Fudan University Shanghai Cancer Center; Approval No. 2006218‐4). The ethics approval numbers for all participating centers are provided in Table S1. Written informed consent was obtained from all participants before enrollment. The trial was performed in accordance with the Declaration of Helsinki, Good Clinical Practice Guidelines, and applicable local regulatory requirements.

## Conflicts of Interest

The authors declare no conflicts of interest.

## Supporting information




**Supporting File**: mco271081‐sup‐0001‐SuppMat.docx.

## Data Availability

All data reported in this paper will be shared by the lead contact upon request. This paper does not report any original code. Trial protocol and any additional information required to reanalyze the data reported in this paper are available from the lead contact upon request.
