# Supplementary material for: Efficacy and Safety of Benmelstobart Plus Anlotinib in Advanced Endometrial Cancer: Results of a Multi‐Cohort, Phase II Study (ETER200)
Source: MedComm (2020). 2026 Oct 21;7(11):e71081. doi: 10.1002/mco2.71081 (PMC13686440; doi:10.1002/mco2.71081)
Supplement: Supplementary file 1 [file MCO2-7-e71081-s001.docx]

**Supporting Information**

**Efficacy and safety of** **benmelstobart plus anlotinib in advanced** **endometrial cancer: results of a multi-cohort, phase II study (ETER200)**

**Running Title:** Benmelstobart plus anlotinib in advanced EC

Shanhui Liang^1 #^, Xiaojun Chen^2,3 #^, Ke Wang^4, #^, Dong Wang^5^, Ruifang An^6^, Jianqing Hou^7^, Ying Yang^7^, Danbo Wang^8^, Pengpeng Qu^9^, Bingzhong Zhang^10^, Wei Duan^11^, Xiumin Li^12^, Aimin Zang^13^, Guohua Yu^14^, Hanmei Lou^15^, Desheng Yao^16^, Shuzhen Wang^17^, Dong Yan^18^, Liang Li^19^, Xiaowei Liu^20^, Weidong Zhao^21^, Zhengzheng Chen^21^, Jing Fei^22^, Jundong Li^23^, Jing Wang^24^, Zhilian Wang^25^, Shuzhong Yao^26^, Fangling Ning^27^, Huaijun Zhou^28^, Li Sun^29^, Lingya Pan^30^, Qing Liu^31^, Haihua Yang^32^, Li Wang^33^, Mingjun Zhang^34^, Xiaoxiang Chen^35^, Jianlin Yuan^36^, Xinwen Zhang^37^, Youzhong Zhang^38^, Qinping Liao^39^, Xiaohua Wu^1, *^

^1^Department of Gynecologic Oncology, Fudan University Shanghai Cancer Center, Shanghai, China

^2^Department of Gynecology and Oncology, Obstetrics & Gynecology Hospital of Fudan University, Shanghai, China

^3^Department of Obstetrics and Gynecology, Shanghai Tenth People's Hospital, Shanghai, China

^4^Department of Gynecologic Oncology, Tianjin Cancer Hospital, Tianjin, China

^5^Gynecologic Oncology Center, Chongqing University Cancer Hospital, Chongqing, China

^6^Department of Obstetrics and Gynecology, First Affiliated Hospital of Xi'an Jiaotong University, Xi'an, China

^7^Department of Gynecology, Yantai Yuhuangding Hospital, Yantai, China

^8^Fifth Department of Obstetrics and Gynecology, Liaoning Cancer Hospital & Institute, Shenyang, China

^9^Department of Gynecology and Oncology, Tianjin Central Hospital of Gynecology Obstetrics, Tianjin, China

^10^Department of Gynecology and Oncology, Sun Yat-Sen Memorial Hospital, Sun Yat-Sen University, Guangzhou, China

^11^Department of Gynecology and Oncology, Beijing Obstetrics and Gynecology Hospital, Capital Medical University, Beijing, China

^12^Department of Gynecology, Linyi Cancer Hospital, Linyi, China

^13^Department of Medical Oncology, Affiliated Hospital of Hebei University, Baoding, China

^14^Department of Oncology, Weifang People's Hospital, Weifang, China

^15^Department of Gynecologic Oncology and Radiation Therapy, Zhejiang Cancer Hospital, Hangzhou, China

^16^Department of Gynecology and Oncology, Guangxi Medical University Cancer Hospital, Nanning, China

^17^Department of Obstetrics and Gynecology, Beijing Chao-Yang Hospital, Capital Medical University, Beijing, China

^18^Department of Oncology, Beijing Luhe Hospital, Capital Medical University, Beijing, China

^19^Department of Oncology, Meizhou People's Hospital, Meizhou, China

^20^Department of Oncology, Affiliated Hospital of Jining Medical University, Jining, China

^21^Department of Obstetrics and Gynecology, The First Affiliated Hospital of USTC, Anhui Provincial Hospital, Hefei, China

^22^Department of Medical Oncology, The First Affiliated Hospital of Shihezi University School of Medicine, Shihezi, China

^23^Department of Gynecology, Sun Yat-Sen University Cancer Center, Guangzhou, China

^24^Fifth Department of Gynecologic Oncology, Hunan Cancer Hospital, Changsha, China

^25^Department of Obstetrics and Gynecology, Second Hospital of Shanxi Medical University, Taiyuan, China

^26^Department of Obstetrics and Gynecology, The First Affiliated Hospital, Sun Yat-Sen University, Guangzhou, China

^27^Department of Oncology, Binzhou Medical University Hospital, Binzhou, China

^28^Department of Obstetrics and Gynecology, Nanjing Drum Tower Hospital, Nanjing, China

^29^Department of Gynecologic Oncology, Qingdao Central Medical Group, Qingdao, China

^30^Department of Obstetrics and Gynecology, Chinese Academy of Medical Sciences and Peking Union Medical College, Beijing, China

^31^Department of Gynecology, Gansu Provincial Maternity and Child-care Hospital, Lanzhou, China

^32^Department of Radiation Oncology, Taizhou Hospital of Zhejiang Province, Taizhou, China

^33^Department of Gynecology and Oncology, Henan Cancer Hospital, Zhengzhou, China

^34^Department of Oncology, The Second Hospital of Anhui Medical University, Hefei, China

^35^Department of Gynecology and Oncology, Jiangsu Cancer Hospital, Nanjing, China

^36^Department of Gynecology, Affiliated Tumor Hospital of Xinjiang Medical University, Urumqi, China

^37^Department of Obstetrics and Gynecology, Xi'an People Hospital (Xi'an Fourth Hospital), Xi'an, China

^38^Department of Gynecology, Qilu Hospital of Shandong University, Jinan, China

^39^Department of Obstetrics and Gynecology, Beijing Tsinghua Changgung Hospital, Beijing, China

^#^These authors contributed equally to this work.

***Corresponding author:** Xiaohua Wu

Department of Gynecologic Oncology, Fudan University Shanghai Cancer Center, No. 270 Dong'an Road, Xuhui District, Shanghai 200032, China

Email: wu.xh@fudan.edu.cn

**Table of Contents**

[**Supplementary Figures**	3](#_Toc236734935)

[Figure S1. Tumor response assessed by IRC per RECIST 1.1 of patients in exploratory stage (n=22)	3](#_Toc236734936)

[Figure S2. Kaplan-Meier plots for duration of response by independent review committee (A) and investigator (B) in the expansion stage (n=85)	4](#_Toc236734937)

[Figure S3. Subgroup analysis of IRC-assessed ORR in the expansion stage (n=85)	5](#_Toc236734938)

[Figure S4. Kaplan-Meier plots for IRC-assessed progression-free survival (A) and overall survival (B) in the overall population (n=107)	6](#_Toc236734939)

[Figure S5. Kaplan-Meier plots for progression-free survival by investigator in the expansion stage (n=85)	7](#_Toc236734940)

[**Supplementary Tables**	8](#_Toc236734941)

[Table S1. List of approval number of ethics committees by participating centers for ETER200	8](#_Toc236734942)

[Table S2. Baseline characteristics of patients in the exploratory stage and overall population	9](#_Toc236734943)

[Table S3. Secondary efficacy endpoints by analysis population	10](#_Toc236734944)

[Table S4. Summary of adverse events in the exploratory stage safety population	11](#_Toc236734945)

[Table S5. Adverse events in the exploratory stage safety population (n=22)	12](#_Toc236734946)

[Table S6. Immune-related adverse events in the exploratory stage safety population (n=22)	13](#_Toc236734947)

[Table S7. ORR and PFS as per RECIST 1.1 according to treatment-free interval in the expansion stage	14](#_Toc236734948)

[Table S8. Summary of drug exposure in the overall safety population	15](#_Toc236734949)

[Table S9. Summary of adverse events in the expansion stage safety population (n=85)	16](#_Toc236734950)

[Table S10. Immune-related adverse events in the expansion stage safety population (n=85)	17](#_Toc236734951)

**Supplementary Figures**

### Figure S1. Tumor response assessed by IRC per RECIST 1.1 of patients in exploratory stage (n=22)

A. Waterfall plot of best percentage change in tumor size from baseline. B. Swimmer plot of time on study and duration of responses. C. Longitudinal percentage change in tumor size from baseline over time.

**Abbreviation:** IRC, independent review committee; RECIST 1.1, Response Evaluation Criteria in Solid Tumors version 1.1. PR, partial response; SD, stable disease; PD, disease progression.

### Figure S2. Kaplan-Meier plots for duration of response by independent review committee (A) and investigator (B) in the expansion stage (n=85)

**Abbreviation:** CI, confidence interval; NE, not evaluable.

### Figure S3. Subgroup analysis of IRC-assessed ORR in the expansion stage (n=85)

**Abbreviation:** IRC, independent review committee; ORR, objective response rate; ECOG, Eastern Cooperative Oncology Group.

### Figure S4. Kaplan-Meier plots for IRC-assessed progression-free survival (A) and overall survival (B) in the overall population (n=107)

**Abbreviation:** IRC, independent review committee; CI, confidence interval.

### Figure S5. Kaplan-Meier plots for progression-free survival by investigator in the expansion stage (n=85)

**Supplementary Tables**

### Table S1. List of approval number of ethics committees by participating centers for ETER200

| **No.** | **Participating centers** | **Approval number** |
| --- | --- | --- |
| 1 | Department of Gynecologic Oncology, Fudan University Shanghai Cancer Center, Shanghai, China | 2006218-4 |
| 2 | Department of Gynecology and Oncology, Obstetrics & Gynecology Hospital of Fudan University, Shanghai, China | 2020-68 |
| 3 | Department of Gynecologic Oncology, Tianjin Cancer Hospital, Tianjin, China | E2020378 |
| 4 | Gynecologic Oncology Center, Chongqing University Cancer Hospital, Chongqing, China | CZLS2020234-A |
| 5 | Department of Obstetrics and Gynecology, First Affiliated Hospital of Xi'an Jiaotong University, Xi'an, China | XJTU1AF2020LSY-92 |
| 6 | Department of Gynecology, Yantai Yuhuangding Hospital, Yantai, China | 2020-32 |
| 7 | Fifth Department of Obstetrics and Gynecology, Liaoning Cancer Hospital & Institute, Shenyang, China | 20200832 |
| 8 | Department of Gynecology and Oncology, Tianjin Central Hospital of Gynecology Obstetrics, Tianjin, China | 2021YS001 |
| 9 | Department of Gynecology and Oncology, Sun Yat-Sen Memorial Hospital, Sun Yat-Sen University, Guangzhou, China | 2020-YW-105-001 |
| 10 | Department of Gynecology and Oncology, Beijing Obstetrics and Gynecology Hospital, Capital Medical University, Beijing, China | 2020-YW-014-01 |
| 11 | Department of Gynecology, Linyi Cancer Hospital, Linyi, China | XY2032 |
| 12 | Department of Medical Oncology, Affiliated Hospital of Hebei University, Baoding, China | HDFY-LL-2020-106 |
| 13 | Department of Oncology, Weifang People's Hospital, Weifang, China | 2020-024-01 |
| 14 | Department of Gynecologic Oncology and Radiation Therapy, Zhejiang Cancer hospital, Hangzhou, China | IRB-[2020]875 |
| 15 | Department of Gynecology and Oncology, Guangxi Medical University Cancer Hospital, Nanning, China | CS2021(54) |
| 16 | Department of Obstetrics and Gynecology, Beijing Chao-Yang Hospital, Capital Medical University, Beijing, China | 2020-YAO-45 |
| 17 | Department of Oncology, Beijing Luhe Hospital, Capital Medical University, Beijing, China | 2020-LHYW-032-01 |
| 18 | Department of Oncology, Meizhou People's Hospital, Meizhou, China | 2022-A-05 |
| 19 | Department of Oncology, Affiliated Hospital of Jining Medical University, Jining, China | (2021)JYFY-18 |
| 20 | Department of Obstetrics and Gynecology, The First Affiliated Hospital of USTC, Anhui Provincial Hospital, Hefei, China | 2020-328 |
| 21 | Department of Medical Oncology, The First Affiliated Hospital of Shihezi University School of Medicine, County-level Administrative Divisions under the Xinjiang Uygur Autonomous Region, China | 2020-005-01 |
| 22 | Department of Gynecology, Sun Yat-Sen University Cancer Center, Guangzhou, China | A2020-121-01 |
| 23 | Fifth Department of Gynecologic Oncology, Hunan Cancer Hospital, Changsha, China | 2020-288 |
| 24 | Department of Obstetrics and Gynecology, Second hospital of Shanxi Medical University, Taiyuan, China | [2020]YW-020 |
| 25 | Department of Obstetrics and Gynecology, The First Affiliated Hospital, Sun Yat-Sen University, Guangzhou, China | 2020-083-01 |
| 26 | Department of Oncology, Binzhou Medical University Hospital, Binzhou, China | 2020-028-01 |
| 27 | Department of Obstetrics and Gynecology, Nanjing Drum Tower Hospital, Nanjing, China | 2020-200-01 |
| 28 | Department of Gynecologic Oncology, Qingdao Central Medical Group, Qingdao, China | [Y]SY202002702 |
| 29 | Department of Obstetrics and Gynecology, Chinese Academy of Medical Sciences and Peking Union Medical College, Beijing, China | KS2020372 |
| 30 | Department of Gynecology, Gansu Provincial Maternity and Child-care Hospital, Lanzhou, China | 20210403 |
| 31 | Department of Radiation Oncology, Taizhou Hospital of Zhejiang Province, Taizhou, China | 202114-01 |
| 32 | Department of Gynecology and Oncology, Henan Cancer Hospital, Zhengzhou, China | 2020051303 |
| 33 | Department of Oncology, The Second Hospital of Anhui Medical University, Hefei, China | YW2020-123 |
| 34 | Department of Gynecology and Oncology, Jiangsu Cancer Hospital, Nanjing, China | 2021-046 |
| 35 | Department of Gynecology, Affiliated Tumor Hospital of Xinjiang Medical University, Urumqi, China | [2020]-033 |
| 36 | Department of Obstetrics and Gynecology, Xi'an People Hospital (Xi'an Fourth Hospital), Xi'an, China | 2021010 |
| 37 | Department of Gynecology, Qilu Hospital of Shandong University, Jinan, China | 2021079 |
| 38 | Department of Obstetrics and Gynecology, Beijing Tsinghua Changgung Hospital, Beijing, China | 20271-1-01 |

### Table S2. Baseline characteristics of patients in the exploratory stage and overall population

| **Characteristics** | **Exploratory stage (n=22)** | **Overall population (n=107)** |
| --- | --- | --- |
| Age, years |  |  |
| <65 years | 16 (72.7) | 79 (73.8) |
| ≥65 years | 6 (27.3) | 28 (26.2) |
| Histology type at diagnosis |  |  |
| Endometrioid carcinoma | 15 (68.2) | 83 (77.6) |
| Serous carcinoma | 5 (22.7) | 21 (19.6) |
| Other | 2 (9.1) | 3 (2.8) |
| ECOG performance status |  |  |
| 0 | 9 (40.9) | 39 (36.4) |
| 1 | 13 (59.1) | 67 (62.6) |
| FIGO stage at diagnosis |  |  |
| I | 8 (36.4) | 29 (27.1) |
| II | 1 (4.5) | 6 (5.6) |
| III | 5 (22.7) | 31 (29.0) |
| IV | 6 (27.3) | 31 (29.0) |
| Disease status |  |  |
| Recurrent | 16 (72.7) | 90 (84.1) |
| Metastatic | 22 (100.0) | 107 (100.0) |
| Metastatic sites |  |  |
| Liver | 8 (36.4) | 35 (32.7) |
| Brain | 1 (4.5) | 5 (4.7) |
| Prior lines of systemic treatment |  |  |
| 1 | 16 (72.7) | 85 (79.4) |
| 2 | 6 (27.3) | 22 (20.6) |
| Prior radiation therapy | 15 (68.2) | 65 (60.7) |
| Prior pelvic surgery | 22 (100.0) | 104 (97.2) |
| Treatment-free interval* |  |  |
| ≤6 months | 13 (59.1) | 64 (59.8) |
| >6 months | 9 (40.9) | 43 (41.2) |

Data are median (range) or n (%).

^*^ Treatment-free interval defined as the interval between the end of the last prior antitumor therapy and the initiation of second-line therapy.

**Abbreviation:** ECOG, Eastern Cooperative Oncology Group; FIGO, International Federation of Gynecology and Obstetrics.

### Table S3. Secondary efficacy endpoints by analysis population

|  | **IRC assessment** | |  | **Investigators assessment** | |
| --- | --- | --- | --- | --- | --- |
| **Efficacy endpoints** | **Exploratory stage (n=22)** | **Overall population (n=107)** |  | **Exploratory stage (n=22)** | **Expansion stage (n=85)** |
| Objective response***** |  |  |  |  |  |
| No. of patients | 8 | 37 |  | 7 | 32 |
| % (95% CI) | 36.4 (17.2-59.3) | 34.6 (25.7-44.4) |  | 31.8 (13.9-54.9) | 37.6 (27.4-48.8) |
| Best response, no. (%) |  |  |  |  |  |
| Complete response | 0 | 2 (1.9) |  | 1 (4.5) | 1 (1.2) |
| Partial response | 8 (36.4) | 35 (32.7) |  | 6 (27.3) | 31 (36.5) |
| Stable disease | 6 (27.3) | 43 (40.2) |  | 8 (36.4) | 34 (40.0) |
| Progressive disease | 7 (31.8) | 20 (18.7) |  | 6 (27.3) | 12 (14.1) |
| Not evaluable^‡^ | 1 (4.5) | 7 (6.5) |  | 1 (4.5) | 7 (8.2) |
| Disease control rate, % (95% CI) | 63.6 (40.7-82.8) | 74.8 (65.5-82.7) |  | 68.2 (45.1-86.1) | 77.6 (67.3-86.0) |

^*^ Analyses based on confirmed response as per RECIST version 1.1.

^§^ Patients without confirmed response or post-baseline tumor assessment.

**Abbreviation:** IRC, Independent review committee; CI, confidence interval.

### Table S4. Summary of adverse events in the exploratory stage safety population

| **Treatment-emergent adverse events** | | **Treatment-related adverse events** | |
| --- | --- | --- | --- |
| **Summary** | **Exploratory stage (n=22)** | **Summary** | **Exploratory stage (n=22)** |
| **Any TEAEs** | 22 (100.0) | **Any TRAEs** | 22 (100.0) |
|  |  | Benmelstobart-related | 19 (86.4) |
|  |  | Anlotinib-related | 22 (100.0) |
| **Grade ≥3 TEAEs** | 17 (77.3) | **Grade ≥3 TRAEs** | 14 (63.6) |
|  |  | Benmelstobart-related | 12 (54.5) |
|  |  | Anlotinib-related | 14 (63.6) |
| **Serious TEAEs** | 10 (45.5) | **Serious TRAEs** | 5 (22.7) |
|  |  | Benmelstobart-related | 2 (9.1) |
|  |  | Anlotinib-related | 5 (22.7) |
| **TEAEs led to dose interruption** | 13 (59.1) | **TRAEs led to dose interruption** | 12 (54.5) |
| Led to benmelstobart interruption | 12 (54.5) | Benmelstobart-related | 8 (36.4) |
| Led to anlotinib interruption | 12 (54.5) | Anlotinib-related | 9 (40.9) |
| **TEAEs led to dose reduction** | 9 (40.9) | **TRAEs led to dose reduction** | 9 (40.9) |
| **TEAEs led to treatment discontinuation** | 3 (13.6) | **TRAEs led to treatment discontinuation** | 2 (9.1) |
| Led to benmelstobart discontinuation | 2 (9.1) | Benmelstobart-related | 1 (4.5) |
| Led to anlotinib discontinuation | 3 (13.6) | Anlotinib-related | 2 (9.1) |

Data are the number (%) of patients with an adverse event.

**Abbreviation:** TEAEs, Treatment-emergent adverse events; TRAEs, Treatment-related adverse events.

### Table S5. Adverse events in the exploratory stage safety population (n=22)

| **Adverse Events, n (%)** | **Treatment-emergent adverse events** | | **Treatment-related adverse events** | |
| --- | --- | --- | --- | --- |
|  | **Any Grade** | **Grade ≥3** | **Any Grade** | **Grade ≥3** |
| Any events | 22 (100.0) | 17 (77.3) | 22 (100.0) | 14 (63.6) |
| Frequent events (≥20% incidence) |  |  |  |  |
| Hypertension | 14 (63.6) | 7 (31.8) | 14 (63.6) | 7 (31.8) |
| Diarrhea | 14 (63.6) | 1 (4.5) | 11 (50.0) | 1 (4.5) |
| Weight loss | 11 (50.0) | 1 (4.5) | 8 (36.4) | 1 (4.5) |
| Blood corticotrophin increased | 11 (50.0) | 0 | 11 (50.0) | 0 |
| TSH increased | 11 (50.0) | 1 (4.5) | 11 (50.0) | 1 (4.5) |
| Hypothyroidism | 10 (45.5) | 0 | 10 (45.5) | 0 |
| White blood cell decreased | 10 (45.5) | 0 | 10 (45.5) | 0 |
| Palmar-plantar erythrodysesthesia syndrome | 8 (36.4) | 0 | 8 (36.4) | 0 |
| AST increased | 7 (31.8) | 1 (4.5) | 7 (31.8) | 1 (4.5) |
| Hypertriglyceridemia | 7 (31.8) | 0 | 6 (27.3) | 0 |
| Hyperthyroidism | 7 (31.8) | 0 | 6 (27.3) | 0 |
| ALT increased | 6 (27.3) | 0 | 6 (27.3) | 0 |
| Cholesterol high | 6 (27.3) | 1 (4.5) | 6 (27.3) | 1 (4.5) |
| Vomiting | 6 (27.3) | 0 | 5 (22.7) | 0 |
| Neutrophil count decreased | 5 (22.7) | 0 | 5 (22.7) | 0 |
| Urinary tract infection | 5 (22.7) | 0 | 4 (18.2) | 0 |
| Proteinuria | 5 (22.7) | 0 | 4 (18.2) | 0 |
| Hypoalbuminemia | 5 (22.7) | 0 | 5 (22.7) | 0 |
| Anorexia | 5 (22.7) | 1 (4.5) | 5 (22.7) | 1 (4.5) |
| Hypokalemia | 5 (22.7) | 0 | 4 (18.2) | 0 |
| Nausea | 5 (22.7) | 0 | 3 (13.6) | 0 |
| Hypocalcemia | 5 (22.7) | 0 | 4 (18.2) | 0 |
| Lipase increased | 5 (22.7) | 1 (4.5) | 5 (22.7) | 1 (4.5) |

Data are the number (%) of patients with an adverse event.

**Abbreviation:** TSH, Thyroid stimulating hormone; AST, Aspartate aminotransferase; ALT, Alanine aminotransferase.

### Table S6. Immune-related adverse events in the exploratory stage safety population (n=22)

|  | **Exploratory stage (n=22)** |
| --- | --- |
| Any irAEs | 12 (54.5) |
| Lead to treatment discontinuation | 0 |
| Lead to benmelstobart interruption | 4 (18.2) |
| Frequent irAEs (≥2% incidence) |  |
| TSH increased | 7 (31.8) |
| Hypothyroidism | 3 (13.6) |
| Diarrhea | 1 (4.5) |
| Rash | 1 (4.5) |
| γ-glutamyltransferase increased | 1 (4.5) |
| AST increased | 1 (4.5) |
| Blood creatinine increased | 1 (4.5) |
| Peripheral neuropathy | 1 (4.5) |
| Adrenal insufficiency | 1 (4.5) |
| Thyroid hormone increased | 1 (4.5) |
| Palpitations | 1 (4.5) |

Data are the number (%) of patients with an adverse event.

**Abbreviation:** irAEs, Immune-related adverse events; TSH, Thyroid stimulating hormone; AST, Aspartate aminotransferase.

### Table S7. ORR and PFS as per RECIST 1.1 according to treatment-free interval in the expansion stage

| **TFI, months** | **Independent review committee assessment (n=85)** | |  | **Investigators assessment (n=85)** | |
| --- | --- | --- | --- | --- | --- |
|  | **ORR % (95% CI)** | **Median PFS (95% CI)** |  | **ORR % (95% CI)** | **Median PFS (95% CI)** |
| ≤6 (n=51) | 31.4 (19.1-45.9) | 6.9 (4.1-9.7) |  | 29.4 (17.5-43.8) | 8.2 (4.2-11.1) |
| >6 (n=34) | 38.2 (22.2-56.4) | 14.2 (9.6-NE) |  | 50.0 (32.4-67.6) | 9.7 (6.7-18.0) |

^*^ Cohort 1: patients with non-MSI-H/pMMR endometrial cancer received benmelstobart plus anlotinib.

**Abbreviation:** TFI, treatment-free interval; ORR, objective response rate; PFS, progression-free survival; CI, confidence interval; NE, not evaluable.

### Table S8. Summary of drug exposure in the overall safety population

|  | **Patients (n=107)^*^** |
| --- | --- |
| **Benmelstobart exposure** |  |
| Median cycles (range) | 11.0 (1.0-49.0) |
| Dose intensity^‡^, mg/cycle |  |
| Mean (SD) | 1179.8 (68.8) |
| Median (IQR) | 1200.0 (1200.0-1200.0) |
| Total cumulative dose^§^, mg |  |
| Mean (SD) | 16900.9 (12998.9) |
| Median (IQR) | 12000.0 (4800.0-25200.0) |
| Relative dose intensity^¶^, % |  |
| Mean (SD) | 98.3 (5.7) |
| Median (IQR) | 100.0 (100.0-100.0) |
| Patients with indicated treatment duration, no.(%) | |
| ≥1 cycles | 107 (100.0) |
| ≥3 cycles | 92 (86.0) |
| ≥6 cycles | 81 (75.7) |
| ≥12 cycles | 53 (49.5) |
| **Anlotinib exposure** |  |
| Median treatment duration (range), day | 152.5 (14.0-770.0) |
| Dose intensity^‡^, mg/day |  |
| Mean (SD) | 11.4 (0.9) |
| Median (IQR) | 12.0 (10.8-12.0) |
| Total cumulative dose^§^, mg |  |
| Mean (SD) | 2319.8 (1945.4) |
| Median (IQR) | 1802.0 (840.0-3360.0) |
| Relative dose intensity^¶^, % |  |
| Mean (SD) | 95.3 (7.2) |
| Median (IQR) | 100.0 (90.0-100.0) |
| Patients with indicated treatment duration, no.(%) | |
| ≥4 weeks | 98 (91.6) |
| ≥8 weeks | 86 (80.4) |
| ≥12 weeks | 78 (72.9) |
| ≥16 weeks | 64 (59.8) |
| ≥20 weeks | 59 (55.1) |
| ≥24 weeks | 51 (47.7) |
| ≥28 weeks | 44 (41.1) |
| ≥32 weeks | 41 (38.3) |
| ≥36 weeks | 38 (35.5) |

^*^ Data of the overall cohort 1 population are shown.

^‡^ Dose intensity was calculated as the cumulative dose divided by the total number of exposure cycles/days.

^§^ Cumulative dose was defined as the sum of the doses administered across all treatment cycles.

^¶^ Relative dose intensity was defined as the actual dose administered divided by the planned dose, multiplied by 100%.

**Abbreviation:** SD, standard deviation; IQR, interquartile range.

### Table S9. Summary of adverse events in the expansion stage safety population (n=85)

| **Treatment-emergent adverse events** | | **Treatment-related adverse events** | |
| --- | --- | --- | --- |
| **Summary** | **Expansion stage (n=85)** | **Summary** | **Expansion stage (n=85)** |
| **Any TEAEs** | 84 (98.8) | **Any TRAEs** | 83 (97.6) |
|  |  | Benmelstobart-related | 80 (94.1) |
|  |  | Anlotinib-related | 83 (97.6) |
| **Grade ≥3 TEAEs** | 66 (77.6) | **Grade ≥3 TRAEs** | 60 (70.6) |
|  |  | Benmelstobart-related | 38 (44.7) |
|  |  | Anlotinib-related | 59 (69.4) |
| **Serious TEAEs** | 35 (41.2) | **Serious TRAEs** | 20 (23.5) |
|  |  | Benmelstobart-related | 12 (14.1) |
|  |  | Anlotinib-related | 19 (22.4) |
| **TEAEs led to dose interruption** | 56 (65.9) | **TRAEs led to dose interruption** | 48 (56.5) |
| Led to benmelstobart interruption | 50 (58.8) | Benmelstobart-related | 38 (44.7) |
| Led to anlotinib interruption | 48 (56.5) | Anlotinib-related | 36 (42.4) |
| **TEAEs led to dose reduction** | 34 (40.0) | **TRAEs led to dose reduction** | 34 (40.0) |
| **TEAEs led to treatment discontinuation** | 10 (11.8) | **TRAEs led to treatment discontinuation** | 9 (10.6) |
| Led to benmelstobart discontinuation | 8 (9.4) | Benmelstobart-related | 4 (4.7) |
| Led to anlotinib discontinuation | 10 (11.8) | Anlotinib-related | 8 (9.4) |

Data are the number (%) of patients with an adverse event.

**Abbreviation:** TEAEs, Treatment-emergent adverse events; TRAEs, Treatment-related adverse events.

### Table S10. Immune-related adverse events in the expansion stage safety population (n=85)

|  | **Expansion stage (n=85)** |
| --- | --- |
| Any irAEs | 50 (58.8) |
| Lead to treatment discontinuation | 2 (2.4) |
| Lead to benmelstobart interruption | 14 (16.5) |
| Frequent irAEs (≥2% incidence) |  |
| TSH increased | 9 (10.6) |
| Hypothyroidism | 31 (36.5) |
| Diarrhea | 4 (4.7) |
| Rash | 4 (4.7) |
| γ-glutamyltransferase increased | 2 (2.4) |
| AST increased | 2 (2.4) |
| Blood creatinine increased | 1 (1.2) |
| Peripheral neuropathy | 1 (1.2) |
| Palmar-plantar erythrodysesthesia syndrome | 4 (4.7) |
| ALT increased | 3 (3.5) |
| Hyperthyroidism | 3 (3.5) |
| Platelet count decreased | 2 (2.4) |
| Free thyroxine decreased | 2 (2.4) |
| Free triiodothyronine decreased | 2 (2.4) |

Data are the number (%) of patients with an adverse event.

**Abbreviation:** irAEs, Immune-related adverse events; TSH, Thyroid stimulating hormone; AST, Aspartate aminotransferase; ALT, Alanine aminotransferase.
